# Computed tomography lung parenchymal descriptions in routine radiological reporting have diagnostic and prognostic utility in patients with idiopathic pulmonary arterial hypertension and pulmonary hypertension associated with lung disease

**DOI:** 10.1183/23120541.00549-2021

**Published:** 2022-01-24

**Authors:** Krit Dwivedi, Robin Condliffe, Michael Sharkey, Robert Lewis, Samer Alabed, Smitha Rajaram, Catherine Hill, Laura Saunders, Peter Metherall, Faisal Alandejani, Dheyaa Alkhanfar, Jim M. Wild, Haiping Lu, David G. Kiely, Andrew J. Swift

**Affiliations:** 1 Dept of Infection, Immunity and Cardiovascular Disease, University of Sheffield, Medical School, Sheffield, UK; 2 Dept of Radiology, Sheffield Teaching Hospitals NHS Trust, Sheffield, UK; 3 Pulmonary Vascular Disease Unit, Royal Hallamshire Hospitals, Sheffield Teaching Hospitals NHS Trust, Sheffield, UK; 4 3DLab, Sheffield Teaching Hospitals NHS Trust, Sheffield, UK; 5 Dept of Computer Science, University of Sheffield, Sheffield, UK; 6 Co-first authors; 7 Co-senior authors

## Abstract

**Background:**

Patients with pulmonary hypertension (PH) and lung disease may pose a diagnostic dilemma between idiopathic pulmonary arterial hypertension (IPAH) and PH associated with lung disease (PH-CLD). The prognostic impact of common computed tomography (CT) parenchymal features is unknown.

**Methods:**

660 IPAH and PH-CLD patients assessed between 2001 and 2019 were included. Reports for all CT scans 1 year prior to diagnosis were analysed for common lung parenchymal patterns. Cox regression and Kaplan–Meier analysis were performed.

**Results:**

At univariate analysis of the whole cohort, centrilobular ground-glass (CGG) changes (hazard ratio, HR 0.29) and ground-glass opacification (HR 0.53) predicted improved survival, while honeycombing (HR 2.79), emphysema (HR 2.09) and fibrosis (HR 2.38) predicted worse survival (all p<0.001). Fibrosis was an independent predictor after adjusting for baseline demographics, PH severity and diffusing capacity of the lung for carbon monoxide (HR 1.37, p<0.05). Patients with a clinical diagnosis of IPAH who had an absence of reported parenchymal lung disease (IPAH-noLD) demonstrated superior survival to patients diagnosed with either IPAH who had coexistent CT lung disease or PH-CLD (2-year survival of 85%, 60% and 46%, respectively, p<0.05). CGG changes were present in 23.3% of IPAH-noLD and 5.8% of PH-CLD patients. There was no significant difference in survival between IPAH-noLD patients with or without CGG changes. PH-CLD patients with fibrosis had worse survival than those with emphysema.

**Interpretation:**

Routine clinical reports of CT lung parenchymal disease identify groups of patients with IPAH and PH-CLD with significantly different prognoses. Isolated CGG changes are not uncommon in IPAH but are not associated with worse survival.

## Abstract 2

**Routine radiological reports describing extent of CT lung parenchymal disease can identify groups of patients with IPAH and PH-CLD with significantly different prognoses** https://bit.ly/3GEjH9n

## Introduction

Pulmonary hypertension (PH) is a heterogeneous, progressive and incurable condition associated with significant morbidity and mortality. Five classification groups with similar clinical and pathological characteristics are described including Group 1 (pulmonary arterial hypertension, PAH) and Group 3 (PH due to chronic lung disease and/or hypoxia, PH-CLD) [1]. PH-CLD most commonly complicates COPD and/or emphysema, interstitial lung disease (ILD) and alveolar hypoventilation [2–5]. Patients with PH-CLD typically present with mild to moderate PH, although a small proportion of patients have severe PH. In contrast patients with idiopathic PAH tend to have more severe PH at presentation. Accurate classification of the form of PH is important as it informs prognosis and impacts on treatment decisions. In PH-CLD the importance of haemodynamically characterising disease severity has also been highlighted [6–8].

In practice, distinguishing between idiopathic pulmonary arterial hypertension (IPAH) and PH-CLD may be challenging [9]. The 6th World Symposium on PH task force recommended that patients with minor lung disease, who otherwise meet criteria for idiopathic disease, may be assigned a diagnosis of IPAH [5]. More recently it has been suggested that patients with mild lung disease but severely abnormal pulmonary haemodynamics (the so-called pulmonary vascular phenotype) [2, 10] are not part of the IPAH continuum but represent a different entity closer to PH-CLD [11]. We have recently demonstrated that the presence of a reduced diffusing capacity of the lung for carbon monoxide (*D*_LCO_) per cent predicted (<45%) is associated with poorer survival and response to PAH therapy in patients diagnosed with IPAH [12], in a carefully phenotyped population where minor lung disease was excluded. Hoeper *et al.* [13] subsequently identified a cluster amongst patients diagnosed with IPAH characterised by older age, frequent comorbidities, a higher proportion of males and a reduced *D*_LCO_.

In addition to pulmonary function assessment, many patients undergoing assessment for suspected PH also undergo chest computed tomography (CT) imaging. We therefore hypothesised that descriptions of lung parenchyma at routine radiological reporting could be used to predict outcomes in patients with IPAH and PH-CLD.

## Methods

### Patient cohort

Patients assigned a diagnosis of IPAH, familial PAH (FPAH) or PH-CLD between February 2001 and January 2019 were identified from the ASPIRE registry (a database of consecutive patients referred to the Sheffield Pulmonary Vascular Diseases Unit). All patients underwent comprehensive multimodality assessment including right heart catheterisation. Patients with PH-CLD associated with conditions other than COPD and/or emphysema or ILD were excluded. Patients with two or more radiological features of possible pulmonary veno-occlusive disease (centrilobular ground-glass opacities (CGG), significant mediastinal lymphadenopathy and interlobular septal lines) were also excluded [14].

### CT analysis

**FIGURE 1:**
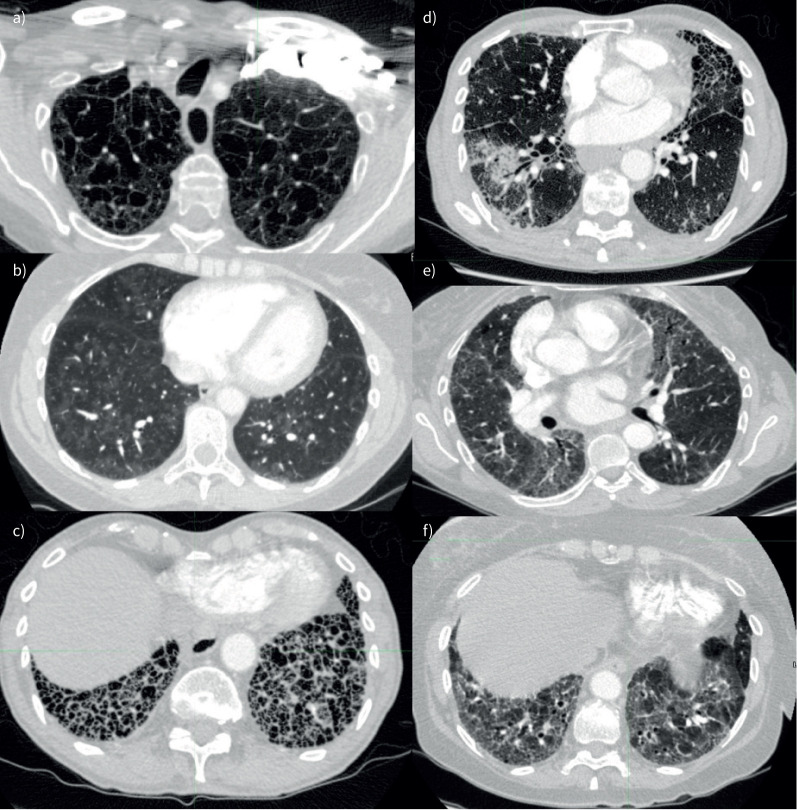
Computed tomography lung parenchymal patterns assessed. a) Emphysema, b) centrilobular ground glass change (windowed to emphasise subtle pattern), c) honeycombing, d) consolidation (with surrounding ground glass change), e) ground glass change and f) fibrosis.

All CT scans, including those performed externally, were reported by specialist cardiothoracic radiologists with expertise in PH using a semi-quantitative assessment of the extent of parenchymal lung disease: none, mild, moderate or severe [15]. Patients had been assigned a diagnosis following a multidisciplinary meeting involving radiologists and pulmonary vascular clinicians. The clinical reports of CT scans performed in the year prior to diagnosis were retrieved. Reports were searched using a regular expression string-search function for mention of six specific lung parenchymal patterns: CGG changes, ground-glass opacification (GGO), honeycombing, consolidation, fibrosis and emphysema. Representative images are shown in figure 1. Each result was manually validated to ensure they represented a true positive. Reports containing false positive phrases such as “no evidence of emphysema” were not counted. The radiologist (K. Dwivedi) who reviewed the CT reports was blinded to the results of other investigations.

### Clinical and mortality data

Clinical data collected included age, sex, World Health Organization (WHO) functional class (FC), pulmonary haemodynamics obtained at right heart catheterisation and pulmonary function tests. Mortality data were obtained from systems linked to the National Health Service Personal Demographics Service (PDS), which is updated when a death is registered in the UK. Patients who emigrated (n=3) were excluded, as were patients without a record on the PDS (n=2). Patients undergoing lung transplantation were censored at the time of surgery, and mortality data were collected using a census date of May 31, 2019.

### IPAH subgroup analysis

Patients with an initial diagnosis of IPAH but with a radiological report of a degree of emphysema or fibrosis were reclassified as “IPAH-lung disease” (IPAH-LD). The remainder of the patients were classified as IPAH-no lung disease (IPAH-noLD). Separate analysis also compared the effect of patients with initial diagnosis of IPAH and CGG changes. Patients with no significant lung disease and CGG were reclassified as “IPAH-CGG”. Survival and group comparison was performed between IPAH-CGG, IPAH-LD and IPAH-noLD. Those with both CGG and significant lung disease (n=8) were excluded from this subgroup analysis.

### Statistics

Analysis was performed with R statistical software package version 4.0.3, using packages “tidyverse” and “survminer”, and SPSS version 26.0 (IBM Corp, Armonk, NY, USA). Continuous data are presented as mean±sd (compared using paired/unpaired t-tests) or median (interquartile range) for nonparametric data (compared using Wilcoxon signed-rank/Mann–Whitney U-tests). Frequencies are compared using the Chi-square test. Categorical variables are shown in magnitude and percentage.

Cox proportional hazard's regression was used to determine association between different CT parenchymal features and survival. Hazard ratios are presented with 95% confidence intervals. Three separate multivariate models were created: model 1 adjusted for patient demographics – age, sex and WHO functional class; model 2 adjusted for all demographics and additionally mean pulmonary arterial pressure (mPAP); and model 3 adjusted further for *D*_LCO_. Kaplan–Meier survival curves were compared using the log-rank test, truncated at 5 years. Group comparisons were made with two-tailed ANOVA with *post hoc* Bonferroni correction.

### Ethics

Ethical approval was granted by the Institutional Review Board and approved by the National Research Ethics Service (16/YH/0352). All research was conducted in agreement with the Declaration of Helsinki and the European General Data Protection Regulation.

## Results

### Patient characteristics

**FIGURE 2:**
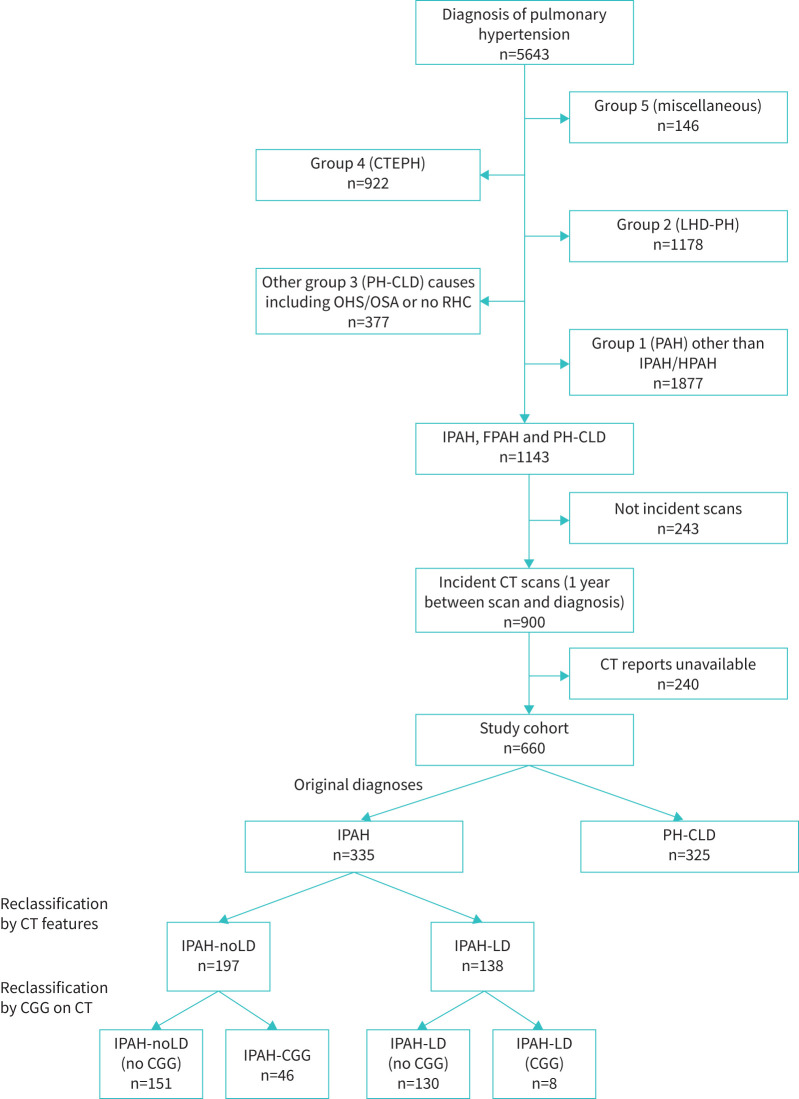
CONSORT (Consolidated Standards of Reporting Trials) flow diagram showing selection of study cohort. CTEPH: chronic thromboembolic pulmonary hypertension; LHD-PH: pulmonary hypertension with left heart disease; PH-CLD: PH due to chronic lung disease and/or hypoxia; OHS: obesity hypoventilation syndrome; OSA: obstructive sleep apnoea; RHC: right heart catheterisation; PAH: pulmonary arterial hypertension; IPAH: idiopathic pulmonary arterial hypertension; FPAH: familial pulmonary arterial hypertension; CT: computed tomography; IPAH-noLD: idiopathic pulmonary arterial hypertension with no lung disease; IPAH-LD: idiopathic pulmonary arterial hypertension with lung disease; CGG: centriobular ground glass.

From 5643 patients diagnosed with all forms of PH, 660 patients met the inclusion criteria and formed the main study cohort (figure 2). This included 335 patients diagnosed with IPAH and 325 with PH-CLD, who formed the subgroups for analysis. Patients with PH-CLD were more frequently male (58% *versus* 39%, p<0.001), older (67±17 years *versus* 60 ±17 years, p<0.001), and had lower mPAP (42±10 mmHg *versus* 53±12 mmHg, p<0.001) and *D*_LCO_ (28±14% *versus* 44±20%, p<0.001) compared to those diagnosed with IPAH (table 1). 283 (43%) patients had imaging performed externally. Right heart catheterisation data were available in 100% and pulmonary function test data in 95% of patients.

**TABLE 1:** Baseline characteristics

**Characteristic**	**Full cohort**	**Subgroups**		**p-value**
		**IPAH**	**PH-CLD**	
**Subjects n**	660	335	325	
**Age at diagnosis years**	64±15	60±17	67±11	<0.001
**Male sex**	318 (48)	131 (39)	187 (58)	<0.001
**WHO functional class**				0.020
II	78 (12)	44 (13)	34 (10)	
III	398 (61)	213 (64)	185 (57)	
IV	181 (28)	76 (23)	105 (32)	
**CT – centrilobular ground-glass (CGG)**	54 (8.2)	54 (16)	0 (0)	<0.001
**CT – ground-glass opacification (GGO)**	93 (14)	62 (19)	31 (9.5)	<0.001
**CT – honeycombing**	15 (2.3)	5 (1.5)	10 (3.1)	0.2
**CT – consolidation**	31 (4.7)	9 (2.7)	22 (6.8)	0.013
**CT – fibrosis**	213 (32)	72 (21)	141 (43)	<0.001
**CT – fibrosis (by severity)**				<0.001
None	447 (68)	263 (79)	184 (57)	
Mild	82 (12)	54 (16)	28 (8.6)	
Moderate	53 (8.0)	9 (2.7)	44 (14)	
Severe	48 (7.3)	0 (0)	48 (15)	
Unknown	30 (4.5)	9 (2.7)	21 (6.5)	
**CT – emphysema**	302 (46)	98 (29)	204 (63)	<0.001
**CT – emphysema (by severity)**				<0.001
None	358 (54)	237 (71)	121 (37)	
Mild	77 (12)	48 (14)	29 (8.9)	
Moderate	129 (20)	38 (11)	91 (28)	
Severe	69 (10)	5 (1.5)	64 (20)	
Unknown	27 (4.1)	7 (2.1)	20 (6.2)	
**CT – CPFE**	101 (15)	32 (9.6)	69 (21)	<0.001
**mPAP mmHg**	47±13	53±12	42±10	<0.001
**mRAP mmHg**	10.2±5.6	11.4±5.8	9.0±5.1	<0.001
**PAWP mmHg**	11.3±3.8	10.8±3.3	11.8±4.2	0.002
**Cardiac output L·min^−1^**	4.65±1.66	4.31±1.62	5.00±1.64	<0.001
**Cardiac index L·min^−1^·m^−2^**	2.53±0.87	2.32±0.81	2.73±0.87	<0.001
**PVR Wood Units**	9.0±5.1	11.0±5.2	7.0±4.1	<0.001
***S*_vO_2__%**	63±9	61±9	65±8	<0.001
**FEV_1_ % predicted**	72±25	83±18	60±25	<0.001
**FEV_1_ severity**				<0.001
Normal (>80% predicted)	259 (41)	187 (58)	72 (24)	
Mild (70–80% predicted)	87 (14)	53 (17)	34 (11)	
Moderate (50–70% predicted)	139 (22)	70 (22)	69 (23)	
Severe (<50% predicted)	140 (22)	11 (3.4)	129 (42)	
**FEV_1_/FVC %**	66±15	71±10	61±18	<0.001
***D*_LCO_ % predicted**	37±19	44±20	28±14	<0.001

Data are presented as n (%) or mean±sd unless otherwise stated. IPAH: idiopathic pulmonary arterial hypertension; PH-CLD: PH due to chronic lung disease and/or hypoxia; CT: computed tomography; WHO: World Health Organisation; CPFE: combined pulmonary fibrosis and emphysema; mPAP: mean pulmonary arterial pressure; mRAP: mean right atrial pressure; PAWP: pulmonary arterial wedge pressure; PVR: pulmonary vascular resistance; *S*_vO_2__: mixed venous oxygen saturation; FEV_1_: forced expiratory volume in 1 s; FVC: forced vital capacity; *D*_LCO_: diffusing capacity of the lung for carbon monoxide.

### Cox regression analysis

Univariate regression results for demographics, clinical parameters and CT features are shown in table 2. Being older and male and having a higher WHO functional class were significant univariate predictors of poor survival across all groups. Table 3 shows the results for the different multivariate regression models performed on significant univariate variables.

**TABLE 2:** Univariate analysis of the overall study cohort

	**Full cohort^#^**			**IPAH^¶^**			**PH-CLD^+^**		
**Characteristic**	**HR**	**95% CI**	**p-value**	**HR**	**95% CI**	**p-value**	**HR**	**95% CI**	**p-value**
**CT – centrilobular ground-glass (CGG)**	0.29	0.17–0.50	**<0.001**	0.44	0.25–0.78	**0.005**			
**CT – ground-glass opacification (GGO)**	0.53	0.38–0.74	**<0.001**	0.52	0.32–0.86	**0.010**	0.72	0.46–1.14	0.2
**CT – honeycombing**	2.79	1.57–4.99	**<0.001**	3.72	1.37–10.1	**0.010**	2.11	1.04–4.30	**0.039**
**CT – consolidation**	0.84	0.50–1.40	0.5	1.10	0.45–2.68	0.8	0.60	0.32–1.13	0.11
**CT – fibrosis**	2.38	1.94–2.91	**<0.001**	2.48	1.76–3.50	**<0.001**	1.83	1.42–2.35	**<0.001**
**CT – fibrosis (any present, ref: none)**									
None	—	—		—	—		—	—	
Mild	1.94	1.46–2.58	**<0.001**	2.51	1.72–3.66	**<0.001**	1.73	1.11–2.71	**0.016**
Moderate	2.77	1.99–3.85	**<0.001**	4.53	2.07–9.92	**<0.001**	1.71	1.18–2.48	**0.005**
Severe	3.19	2.30–4.43	**<0.001**				1.98	1.40–2.80	**<0.001**
**CT – emphysema**	2.09	1.71–2.56	**<0.001**	2.74	1.96–3.81	**<0.001**	1.13	0.87–1.47	0.4
**CT – emphysema (any present, ref: none)**									
None	—	—		—	—		—	—	
Mild	1.78	1.30–2.43	**<0.001**	2.90	1.89–4.46	**<0.001**	0.89	0.56–1.42	0.6
Moderate	2.18	1.69–2.81	**<0.001**	3.16	2.01–4.97	**<0.001**	1.13	0.82–1.55	0.5
Severe	2.92	2.15–3.97	**<0.001**	11.1	3.92–31.6	**<0.001**	1.37	0.97–1.93	0.075
**CT – CPFE**	2.20	1.72–2.80	**<0.001**	2.09	1.33–3.29	**0.001**	1.82	1.36–2.44	**<0.001**
**Age at diagnosis years**	1.05	1.04–1.05	**<0.001**	1.06	1.05–1.08	**<0.001**	1.02	1.01–1.04	**<0.001**
**Male sex**	1.66	1.36–2.03	**<0.001**	1.59	1.16–2.18	**0.004**	1.42	1.10–1.83	**0.007**
**WHO functional class III and IV (ref: I and II)**	1.78	1.45–2.18	**<0.001**	1.88	1.34–2.64	**<0.001**	1.74	1.35–2.25	**<0.001**
**WHO functional class**									
II	—	—		—	—		—	—	
III	2.57	1.70–3.89	**<0.001**	3.00	1.51–5.96	**0.002**	2.45	1.46–4.13	**<0.001**
IV	5.08	3.31–7.79	**<0.001**	5.49	2.69–11.2	**<0.001**	4.80	2.81–8.21	**<0.001**
**mPAP mmHg**	0.99	0.98–1.00	**0.028**	0.98	0.97–0.99	**0.006**	1.04	1.03–1.05	**<0.001**
**mRAP mmHg**	1.01	0.99–1.03	0.4	1.03	1.00–1.06	**0.023**	1.03	1.00–1.05	0.057
**PAWP mmHg**	1.02	0.99–1.04	0.3	1.03	0.98–1.09	0.2	0.98	0.95–1.01	0.2
**Cardiac output L·min^−1^**	0.92	0.86–0.98	**0.009**	0.88	0.78–0.99	**0.035**	0.79	0.72–0.86	**<0.001**
**Cardiac index L·min^−1^·m^−2^**	0.87	0.76–0.98	**0.028**	0.80	0.63–1.01	**0.063**	0.61	0.51–0.74	**<0.001**
**PVR (Wood Units)**	1.04	1.00–1.08	**0.036**	0.98	0.91–1.05	0.5	1.10	1.05–1.16	**<0.001**
***S*_vO_2__%**	0.98	0.97–0.99	**0.001**	0.97	0.95–0.98	**<0.001**	0.96	0.94–0.97	**<0.001**
**FEV_1_ % predicted**	1.00	0.99–1.00	**0.032**	0.99	0.98–1.00	0.11	1.01	1.01–1.02	**<0.001**
**FVC % predicted**	1.00	0.99–1.00	**0.028**	1.00	0.99–1.01	0.9	1.00	1.00–1.01	0.4
**FEV_1_/FVC ratio**	0.99	0.99–1.00	**0.038**	0.97	0.95–0.98	**<0.001**	1.02	1.01–1.03	**<0.001**
***D*_LCO_ % predicted**	0.95	0.95–0.96	**<0.001**	0.95	0.94–0.96	**<0.001**	0.96	0.95–0.97	**<0.001**

Bold text: meets statistical significance. IPAH: idiopathic pulmonary arterial hypertension; PH-CLD: PH due to chronic lung disease and/or hypoxia; HR: hazard ratio; CI: confidence interval; CT: computed tomography; WHO: World Health Organisation; CPFE: combined pulmonary fibrosis and emphysema; mPAP: mean pulmonary arterial pressure; mRAP: mean right atrial pressure; PAWP: pulmonary arterial wedge pressure; PVR: pulmonary vascular resistance; *S*_vO_2__: mixed venous oxygen saturation; FEV_1_: forced expiratory volume in 1 s; FVC: forced vital capacity; *D*_LCO_: diffusing capacity of the lung for carbon monoxide. ^#^: n=660; ^¶^: n=335; ^+^: n=325.

**TABLE 3:** Multivariate analysis

**Characteristic**	**Univariate**			**Multivariate 1 (adjusted for demographics – age, sex, WHO FC)**			**Multivariate 2 (adjusted for demographics and mPAP)**			**Multivariate 3** (**adjusted for demographics, mPAP and *D***_**LCO**_**)**		
	**HR**	**95% CI**	**p-value**	**HR**	**95% CI**	**p-value**	**HR**	**95% CI**	**p-value**	**HR**	**95% CI**	**p-value**
**Full cohort (n=660)**												
CT – centrilobular ground-glass (CGG)	0.29	0.17–0.50	**<0.001**	0.50	0.28–0.89	**0.010**	0.48	0.26–0.86	**0.007**	0.76	0.39–1.47	0.4
CT – ground-glass opacification (GGO)	0.53	0.38–0.74	**<0.001**	0.82	0.58–1.16	0.2	0.84	0.60–1.19	0.3	0.80	0.54–1.18	0.2
CT – honeycombing	2.79	1.57–4.99	**<0.001**	1.73	0.97–3.11	**0.087**	1.74	0.97–3.12	0.086	1.10	0.54–2.24	0.8
CT – emphysema	2.09	1.71–2.56	**<0.001**	1.48	1.21–1.83	**<0.001**	1.52	1.23–1.88	**<0.001**	1.13	0.89–1.44	0.3
CT – fibrosis	2.38	1.94–2.91	**<0.001**	1.75	1.42–2.15	**<0.001**	1.77	1.43–2.18	**<0.001**	1.37	1.09–1.73	**0.008**
**IPAH (n=335)**												
CT – centrilobular ground-glass (CGG)	0.44	0.25–0.78	**0.005**	0.79	0.43–1.45	0.4	0.80	0.43–1.48	0.5	0.92	0.47–1.82	0.8
CT – ground-glass opacification (GGO)	0.52	0.32–0.86	**0.010**	0.88	0.52–1.47	0.6	0.91	0.54–1.52	0.7	0.96	0.55–1.69	0.9
CT – honeycombing	3.72	1.37–10.1	**0.010**	1.69	0.61–4.65	0.3	1.68	0.61–4.62	0.4	1.35	0.49–3.74	0.6
CT – emphysema	2.74	1.96–3.81	**<0.001**	1.72	1.22–2.42	**0.002**	1.76	1.24–2.49	**0.002**	1.26	0.85–1.86	0.2
CT – fibrosis	2.48	1.76–3.50	**<0.001**	1.42	0.99–2.02	0.060	1.44	1.00–2.07	0.056	1.23	0.84–1.81	0.3
**PH-CLD (n= 325)**												
CT – honeycombing	2.11	1.04–4.30	**0.039**	1.80	0.88–3.67	0.14	2.02	0.99–4.14	0.081	1.06	0.39–2.91	>0.9
CT – fibrosis	1.83	1.42–2.35	**<0.001**	1.73	1.34–2.24	**<0.001**	1.63	1.26–2.10	**<0.001**	1.46	1.09–1.96	**0.011**

Bold text: meets statistical significance. WHO FC: World Health Organization functional class; mPAP: mean pulmonary arterial pressure; *D*_LCO_: diffusing capacity of the lung for carbon monoxide; HR: hazard ratio; CI: confidence interval; CT: computed tomography; IPAH: idiopathic pulmonary arterial hypertension; PH-CLD: pulmonary hypertension associated with chronic lung disease.

#### Main cohort

CGG changes (HR 0.29, 95% CI 0.17–0.50) and GGO (HR 0.53, 95% CI 0.38–0.74) were significant (p<0.001) predictors of improved survival at univariate analysis, while honeycombing (HR 2.79, 95% CI 1.57–4.99), emphysema (HR 2.09, 95% CI 1.71–2.56) and fibrosis (HR 2.38, 95% CI 1.94–2.91) were significant predictors of poor survival (all p<0.001). After adjusting for the impact of age, sex and WHO FC in multivariate model 1, CGG changes (HR 0.50, 95% CI 0.28–0.89, p=0.01), emphysema (HR 1.48, 95% CI 1.21–1.83, p<0.001) and fibrosis (HR 1.75, 95% CI 1.42–2.15, p<0.001) remained significant predictors of outcome. These parameters were also significant predictors of mortality in model 2 after additionally adjusting for the severity of PH. In model 3, fibrosis (HR 1.37, 95% CI 1.09–1.73, p=0.008) remained an independent predictor after additionally adjusting for *D*_LCO_.

#### IPAH

In patients assigned a diagnosis of IPAH, the presence of CGG changes (HR 0.44, 95% CI 0.25–0.78, p=0.005) or GGO (HR 0.52, 95% CI 0.32–0.86, p=0.01) predicted improved outcomes, while the presence of any degree of emphysema (HR 2.74, 95% CI 1.96–3.81, p<0.001), fibrosis (HR 2.48, 95% CI 1.76–3.50, p<0.001) or honeycombing (HR 3.72, 95% CI 1.37–10.1, p=0.01) were significant univariate predictors of increased mortality. Subgroup univariate analysis of IPAH patients with no degree of parenchymal lung disease demonstrated that CGG changes and GGO no longer significantly predicted survival (supplementary table A1). In multivariate model 1 of the overall IPAH group, only the presence of emphysema (HR 1.72, 95% CI 1.22–2.42, p=0.002) remained a significant predictor. Emphysema was also a significant prognostic factor in model 2 (HR 1.76, 95% CI 1.24–2.49, p=0.002) but not in model 3 (p=0.3).

#### PH-CLD

Fibrosis (HR 1.83, 95% CI 1.04–4.30, p<0.001) and honeycombing (HR 2.11, 95% CI 1.04–4.30, p=0.039) were significant predictors of mortality at univariate analysis, while the presence of emphysema or GGO did not predict mortality. However, when subgrouped by radiologically graded severity, severe emphysema was a significant prognostic factor at univariate (HR 1.81, p=0.02) but not multivariate analysis. Fibrosis was an independent predictor in all multivariate models, including after adjustment for *D*_LCO_ (Model 3, HR 1.46, 95% CI 1.09–1.96, p<0.001). Honeycombing was not a significant predictor in any of the multivariate models.

#### Prognostic effect of extent of emphysema and fibrosis

Increasing extent of both emphysema and fibrosis derived from radiology reports was associated with increasing risk of mortality in the whole cohort at univariate analysis (emphysema – mild: HR 1.78 (95% CI 1.3–2.43), moderate: HR 2.18 (95% CI 1.69–2.81), severe: HR 2.92 (95% CI 2.15–3.97); fibrosis – mild: HR 1.94 (95% CI 1.46–2.58), moderate: 2.77 (95% CI 1.99–3.85), severe: 3.19 (95% CI 2.3–4.43), table 2). A similar pattern was observed in the IPAH group, while in the PH-CLD group the presence of emphysema was not associated with significant increased mortality.

### Kaplan–Meier survival analysis stratified by CT features

**FIGURE 3:**
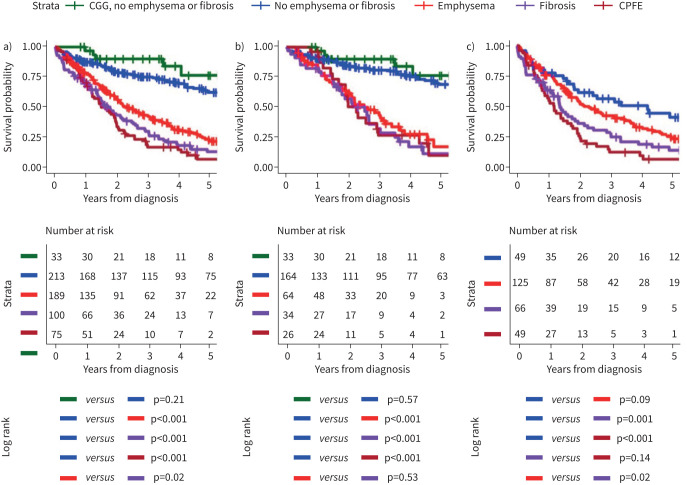
Kaplan–Meier survival curves stratified by computed tomography features of CGG, emphysema and fibrosis for: a) all patients, b) patients initially diagnosed with IPAH and c) patients initially diagnosed with PH-CLD. IPAH: idiopathic pulmonary arterial hypertension; PH-CLD: PH due to chronic lung disease and/or hypoxia; CGG: centrilobular ground-glass; CPFE: combined pulmonary fibrosis and emphysema.

The Kaplan–Meier survival curves for each cohort are presented in figure 3. In the full cohort, survival was significantly worse in patients with any form of parenchymal lung disease (all p<0.001). Survival was also significantly worse in patients with reported fibrosis compared to emphysema (p=0.023). There were 7 (of 33), 85 (of 213), 131 (of 189), 79 (of 100) and 63 (of 75) events in the CGG and no emphysema or fibrosis, no emphysema or fibrosis, emphysema, fibrosis and combined emphysema and fibrosis groups, respectively.

In patients diagnosed with IPAH, the presence of any parenchymal lung disease was associated with significantly poorer survival (all p<0.001) with no significant difference in survival between patients with the different forms of lung disease. There was no significant difference in survival between patients with isolated CGG compared to those with no reported parenchymal abnormalities (p=0.57). There were 7 (of 33), 55 (of 164), 41 (of 64), 26 (of 34) and 18 (of 26) events in the CGG and no emphysema or fibrosis, no emphysema or fibrosis, emphysema, fibrosis and combined emphysema and fibrosis groups, respectively.

In patients diagnosed with PH-CLD, survival in patients with emphysema was superior to survival in patients with fibrosis (p=0.02). Although some evidence of improved survival was observed between patients without parenchymal lung disease compared to those with emphysema, differences between groups did not meet conventional levels of statistical significance (p=0.09). There were 30 (of 49), 90 (of 125), 53 (of 66) and 45 (of 49) in the no emphysema or fibrosis, emphysema, fibrosis and combined emphysema and fibrosis groups, respectively.

### IPAH subgroup analysis

#### Impact of lung disease in IPAH compared to PH-CLD

**FIGURE 4:**
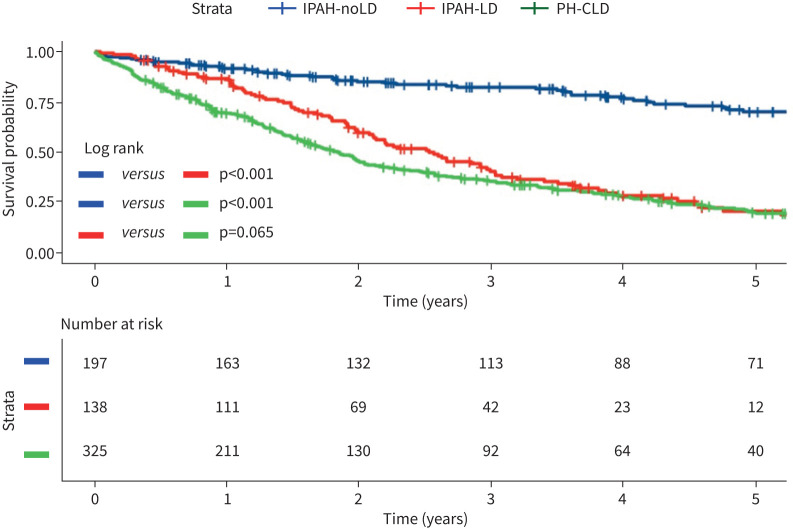
Kaplan–Meier survival curves for patients classified as IPAH-LD, IPAH-noLD and PH-CLD. IPAH-noLD: idiopathic pulmonary arterial hypertension with no CT features of lung disease; IPAH-LD: idiopathic pulmonary arterial hypertension with CT features of lung disease; PH-CLD: pulmonary hypertension associated with chronic lung disease.

Patients with an initial clinical diagnosis of IPAH (n=335) were firstly reclassified as either IPAH-LD (n=138) or IPAH-noLD (n=197) based on the presence or absence of emphysema and/or fibrosis. Survival in patients with IPAH-noLD was significantly superior to patients with IPAH-LD or PH-CLD (p<0.001, figure 4). There were 62 (of 197), 92 (of 138) and 247 (of 325) events in the IPAH, IPAH-LD and PH-CLD groups, respectively. Patients with IPAH-LD were distinct from those diagnosed with PH-CLD with less severely impaired spirometry but more severely abnormal pulmonary haemodynamics (supplementary table A2). There was no statistically significant difference in survival (p=0.065) in patients with PH-CLD when compared to IPAH-LD. Two-year survival for patients with IPAH-noLD, IPAH-LD and PH-CLD was 85%, 60% and 46%, respectively (supplementary table A3).

#### Impact of CGG

**FIGURE 5:**
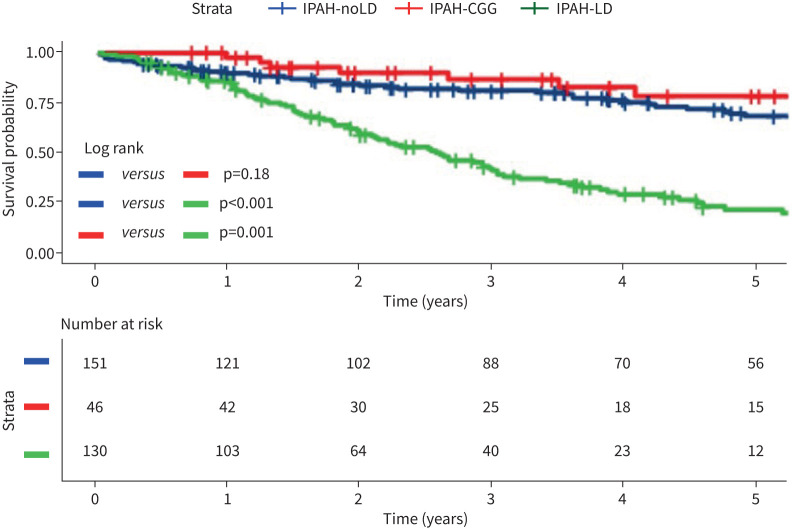
Kaplan–Meier curve comparing survival in IPAH-noLD, IPAH-LD and IPAH-CGG. IPAH-noLD: idiopathic pulmonary arterial hypertension with no computed tomography (CT) features of lung disease; IPAH-LD: idiopathic pulmonary arterial hypertension with CT features of lung disease; IPAH-CGG: idiopathic pulmonary arterial hypertension with centrilobular ground-glass changes.

The impact of CGG changes on characteristics and survival of patients with an initial clinical diagnosis of IPAH (n=335) was then assessed. CGG changes were uncommon in patients with coexisting lung disease (being present in 8 out of 138 patients (5.8%) with IPAH-LD, supplementary table A2) compared with patients with no coexisting lung disease (being present in 46 out of 197 patients (23.3%), table 4). Eight patients who had both CGG changes and parenchymal lung disease were excluded from this analysis. 130 patients with emphysema or fibrosis were therefore reclassified as IPAH-LD, 46 patients with CGG changes as IPAH-CGG and 151 patients with no parenchymal abnormalities as IPAH-noLD. Patients with IPAH-CGG had more severe PH but a similar *D*_LCO_ when compared with patients with IPAH-noLD (table 4). Patients with IPAH-LD were older, with a greater proportion in WHO FC IV and less severe PH but a lower *D*_LCO_ than patients with IPAH-noLD. There was no significant difference in survival between patients with IPAH-noLD and IPAH-CGG, while survival in patients with IPAH-LD was significantly worse (figure 5).

**TABLE 4:** Baseline characteristics of patients with initial diagnosis of IPAH

**Characteristic**	**IPAH-noLD**	**IPAH-CGG**	**IPAH-LD**	**p-value**
**Subjects n**	151	46	130	
**Age at diagnosis**	56±18^#^	48±19^#^	70±9^¶,+^	<0.001
**Male sex**	48 (32)^#^	11 (24)^#^	69 (53)^¶,+^	<0.001
**WHO functional class**	^#^	^#^	^¶,+^	0.011
II	25 (17)	9 (20)	9 (6.9)	
III	98 (65)	29 (64)	81 (62)	
IV	27 (18)	7 (16)	40 (31)	
**Smoker, ever**	66 (49)^#^	19 (42)^#^	81 (86)^¶,+^	<0.001
**CT – centrilobular ground-glass**	0 (0)^+^	46 (100)^¶,#^	0 (0)^+^	<0.001
**CT – ground-glass opacification**	9 (6.0)^+^	39 (85)^¶,#^	8 (6.2)^+^	<0.001
**CT – honeycombing**	0 (0)^#^	0 (0)^¶,#^	5 (3.8)^¶^	0.021
**CT – consolidation**	5 (3.3)	2 (4.3)	2 (1.5)	0.4
**CT – fibrosis**	0 (0)^#^	0 (0)^#^	68 (52)^¶,+^	<0.001
**CT – fibrosis (by severity)**	^#^	^#^	^¶,+^	<0.001
None	151 (100)	46 (100)	62 (48)	
Mild	0 (0)	0 (0)	52 (40)	
Moderate	0 (0)	0 (0)	9 (6.9)	
Unknown	0 (0)	0 (0)	7 (5.4)	
**CT – emphysema**	0 (0)^#^	0 (0)^#^	94 (72)^¶,+^	<0.001
**CT – emphysema (by severity)**	^#^	^#^	^¶,+^	
None	151 (100)	46 (100)	36 (28)	
Mild	0 (0)	0 (0)	44 (34)	
Moderate	0 (0)	0 (0)	38 (29)	
Severe	0 (0)	0 (0)	5 (3.8)	
Unknown	0 (0)	0 (0)	7 (5.4)	
**CT – CPFE**	0 (0)^#^	0 (0)^#^	32 (25)^¶,+^	<0.001
**mPAP mmHg**	54±12^+,#^	62±13^¶,#^	49±8^¶,+^	<0.001
**mRAP mmHg**	12±6	10±5	11±5	0.3
**PAWP mmHg**	10.91±3.10	9.72±2.77	11.03±3.21	0.055
**Cardiac output L·min^−1^**	4.64±1.87^+,#^	3.85±0.98^¶^	4.08±1.44^¶^	0.014
**Cardiac index L·min^−1^·m^−2^**	2.47±0.95^#^	2.13±0.52	2.23±0.73^¶^	0.031
**PVR Wood Units**	10.7±5.2^+^	14.6±6.2^¶,#^	10.3±4.4^+^	<0.001
***S*_vO_2__%**	61±10	62±7	59±8	0.030
**FEV_1_ % predicted**	82±17	88±15	83±20	0.124
**FEV_1_ severity**				0.056
Normal (>80% predicted)	83 (58)	31 (70)	67 (53)	
Mild (70–80% predicted)	21 (15)	8 (18)	24 (19)	
Moderate (50–70% predicted)	34 (24)	5 (11)	29 (23)	
Severe (<50% predicted)	5 (3.5)	0 (0)	6 (4.8)	
**FVC % predicted**	93±20	101±18	99±21	0.016
**FEV_1_/FVC %**	74±10^#^	74±8^#^	67±10^¶,+^	<0.001
***D*_LCO_ % predicted**	52±20^#^	56±17^#^	31±14^¶,+^	<0.001

Data are presented as n (%) or mean±sd unless otherwise stated. Between-group comparisons performed using one-way ANOVA with Bonferroni *post hoc* correction. Eight patients with both CGG and LD not included. IPAH: idiopathic pulmonary arterial hypertension; IPAH-noLD: idiopathic pulmonary arterial hypertension with no computed tomography features of lung disease; IPAH-CGG: idiopathic pulmonary arterial hypertension with centrilobular ground-glass changes; IPAH-LD: idiopathic pulmonary arterial hypertension with CT features of lung disease; CT: computed tomography; WHO: World Health Organisation; CPFE: combined pulmonary fibrosis and emphysema; mPAP: mean pulmonary arterial pressure; mRAP: mean right atrial pressure; PAWP: pulmonary arterial wedge pressure; PVR: pulmonary vascular resistance; *S*_vO_2__: mixed venous oxygen saturation; FEV_1_: forced expiratory volume in 1 s; FVC: forced vital capacity; *D*_LCO_: diffusing capacity of the lung for carbon monoxide; CGG: centrilobular ground-glass; LD: lung disease. Difference between groups noted – ^#^: significant difference to IPAH-LD; ^¶^: significant difference to IPAH-noLD; ^+^: significant difference to IPAH-CGG.

## Discussion

We have demonstrated that parenchymal abnormalities noted at routine radiological reporting of CT scans performed in patients with suspected IPAH or PH-CLD have diagnostic and prognostic utility. We have also found that isolated CGG changes are not uncommon in patients with IPAH and are not associated with lower *D*_LCO_ or worse survival.

### Routine radiological reports of the presence of emphysema or fibrosis have diagnostic and prognostic utility in patients diagnosed with IPAH

Differentiating PH-CLD from IPAH is an important part of the PH diagnostic algorithm and may be straightforward in the presence of severe lung function abnormalities or severe parenchymal lung disease, although in this study we still identified five patients (with mPAP of 55 mmHg) with severe emphysema who had been assigned a clinical diagnosis of IPAH. The presence of less severe degrees of lung disease provides more of a diagnostic challenge as has been highlighted by others [9, 11]. The 6th World Symposium on Pulmonary Hypertension (WSPH) task force suggested that patients with significant PH but only modest parenchymal abnormalities should be assigned a diagnosis of IPAH [5].

In the present study we found that radiological reports of emphysema or fibrosis in patients who had been clinically assigned a diagnosis of IPAH in keeping with the 6th WSPH recommendations was associated with significantly worse survival at Kaplan–Meier analysis. This supports the comments of Godinas *et al.* [11] who suggest that such patients represent a distinct group from those with IPAH being phenotypically closer to PH-CLD.

However, we also observed at Cox regression analysis that this prognostic importance of emphysema or fibrosis in patients diagnosed with IPAH was not independent of *D*_LCO_. The prognostic importance of reduced *D*_LCO_ with or without coexisting parenchymal lung disease in patients assigned a diagnosis of IPAH has previously been reported [15–17]. We have previously demonstrated that response to PAH therapies in patients with IPAH-noLD and *D*_LCO_ <45% is lower than in patients with IPAH-noLD and *D*_LCO_ ≥45%, while on average patients with IPAH-LD exhibit a lack of response to PAH therapies in terms of exercise capacity and quality of life [15]. Therefore, the identification of emphysema or fibrosis on CT provides clinically relevant information in addition to that provided by *D*_LCO_ alone.

### Routine radiological reports of the extent and nature of parenchymal lung disease have prognostic utility

In addition to the presence of parenchymal lung disease identified at routine reporting being associated with worse survival, we also observed that the extent and nature of lung disease described at routine reporting provided additional prognostic information. In patients originally assigned a diagnosis of IPAH, the extent of emphysema or fibrosis described qualitatively at reporting was strongly associated with prognosis. In patients with PH-CLD, the nature of parenchymal lung disease impacted on survival with the presence of fibrosis conveying a worse prognosis than emphysema, as previously described [18].

### CGG in IPAH

CGG changes in the absence of emphysema or fibrosis were not uncommon, being reported in 23.3% of those IPAH patients who had no emphysema or fibrosis but were only reported in only 5.8% of patients with coexisting emphysema or fibrosis. The nature of CGG changes in patients diagnosed with IPAH is not clear. It is possible they represent pulmonary veno-occlusive disease (PVOD); however, we excluded patients with an additional radiological feature of PVOD (significant mediastinal lymphadenopathy or interlobular septal lines). Furthermore, there was no significant difference in *D*_LCO_ compared to IPAH-noLD patients without CGG changes. Nolan *et al.* [19] postulated that they represented cholesterol granulomas, while Horton *et al.* [20] presented a case report of a patient with fenfluramine-induced PAH with diffuse micronodules on CT who had an extensive diffuse plexogenic arteriopathy at lung biopsy [20]. In addition to IPAH, CGG changes have been commonly described in other forms of PAH including PAH associated with connective tissue disease and congenital heart disease [21]. In Eisenmenger syndrome and IPAH it has been postulated that this may be a feature of lung neovascularity [22, 23]. It is interesting to note that in our study there was no significant difference in survival compared with patients with IPAH-noLD at Kaplan–Meier analysis despite having significantly more severe pulmonary haemodynamics.

### Limitations

This study has utilised clinical radiological reports involving semi-quantitative descriptions from the time of PH diagnosis rather than subsequent quantitative analysis. By using this approach, we have, however, been able to demonstrate that features described in routine “real-world” radiological reports have the ability to refine diagnostic and prognostic processes. It may well be that more in-depth quantitative analysis utilising artificial intelligence algorithms may provide superior diagnostic and prognostic information, and further studies are therefore warranted [24]. Selection or recall bias is minimised by using a “real-world” clinical cohort of consecutive patients diagnosed over 18 years. Misclassification bias is minimised by including all consecutive patients with lung disease and PH, not limiting to just an assigned diagnosis. An unavoidable limitation of a clinical cohort spanning 18 years is the variance in haemodynamic diagnostic criteria, treatment options and guidelines for patient management.

### Conclusion

CT lung parenchymal descriptions in routine radiological reporting have diagnostic and prognostic utility in patients with IPAH and PH associated with chronic lung disease. Chest CT features should therefore be considered in patient assessment and risk stratification. Patients diagnosed with IPAH who have modest lung disease demonstrate unique clinical and survival characteristics and are likely to represent a distinct phenotype separate from IPAH. CGG changes are not uncommon in patients with IPAH and are associated with more severe pulmonary haemodynamics but non-inferior survival.

## Supplementary material

10.1183/23120541.00549-2021.Supp1 **Please note:** supplementary material is not edited by the Editorial Office, and is uploaded as it has been supplied by the author.
Supplementary material 00549-2021.SUPPLEMENT
