# Supplementary material for: Computed tomography lung parenchymal descriptions in routine radiological reporting have diagnostic and prognostic utility in patients with idiopathic pulmonary arterial hypertension and pulmonary hypertension associated with lung disease
Source: ERJ Open Res. 2022 Jan 24;8(1):00549-2021. doi: 10.1183/23120541.00549-2021 (PMC8784758; doi:10.1183/23120541.00549-2021)
Supplement: Supplementary file 1 [file 00549-2021.SUPPLEMENT.pdf]

Appendix

Table A1: Univariate analysis of patients with IPAH-noLD

**Characteristic**	**HR**[1]	**95% CI**[1]	**p-value**
CT - Centrilobular Ground Glass (CGG)	0.62	0.30, 1.26	0.2
CT - Ground Glass Opacification (GGO)	0.64	0.32, 1.26	0.2
CT - Honeycombing			
CT - Consolidation	1.55	0.48, 4.96	0.5
Age at diagnosis	1.05	1.03, 1.07	<0.001
Male Gender	1.50	0.90, 2.52	0.12
WHO Functional Class III & IV (ref: I & II)	1.78	1.12, 2.81	0.014
WHO Functional Class			
II	—	—	
III	2.61	1.02, 6.67	0.046
IV	5.62	2.07, 15.2	<0.001
mPAP (mmHg)	0.99	0.97, 1.01	0.3
mRAP (mmHg)	1.04	1.00, 1.08	0.085
PAWP (mmHg)	1.10	1.00, 1.20	0.040
Cardiac output (L/min)	1.00	0.84, 1.18	>0.9
Cardiac index (L/min/m^2)	0.98	0.70, 1.38	>0.9
PVR (Wood Units)	0.97	0.92, 1.02	0.2
SvO_2 %	0.96	0.94, 0.99	0.011
FEV_1 % predicted	0.98	0.97, 0.99	0.008
FVC % predicted	0.99	0.98, 1.00	0.11
FEV_1/FVC Ratio	0.96	0.94, 0.98	<0.001
DLco % predicted	0.95	0.93, 0.97	<0.001

Abbreviations: HR - Hazard Ratio, CI - Confidence Interval, IPAH-noLD, Idiopathic Pulmonary Arterial Hypertension with no lung disease, CT – Computed Tomography, WHO – World Health Organisation, CPFE – Combined Pulmonary Fibrosis and Emphysema, mPAP – mean pulmonary arterial pressure, mRAP – mean right atrial pressure, PAWP – pulmonary arterial wedge pressure, PVR – pulmonary vascular resistance, SvO2 – mixed venous oxygen saturation. FEV_1 – forced expiratory volume in 1 second, FVC – forced vital capacity, DLco – diffusing capacity of carbon monoxide.

Table A2: Baseline characteristics of IPAH-noLD vs IPAH-LD vs PH-CLD

Characteristic	IPAH-noLD, N = 197	IPAH-LD, N = 138	PH-CLD, N = 325	p-value
Age at diagnosis	54 (18) †‡	70 (10) *	67 (11) *	<0.001
Male Gender	59 (30%)†‡	72 (52%)*	187 (58%)*	<0.001
WHO Functional Class	†‡	*	*	<0.001
2	34 (17%)†‡	10 (7.2%)*	34 (10%)	
3	127 (65%)	86 (62%)	185 (57%)	
4	34 (17%)	42 (30%)	105 (32%)	
CT - Centrilobular Ground Glass (CGG)	46 (23%)†‡	8 (5.8%)*	0 (0%)*	<0.001
CT - Ground Glass Opacification (GGO)	48 (24%)†‡	14 (10%)*	31 (9.5%)*	<0.001
CT - Honeycombing	0 (0%)	5 (3.6%)	10 (3.1%)	0.013
CT - Consolidation	7 (3.6%)	2 (1.4%)‡	22 (6.8%)†	0.031
CT - Fibrosis	0 (0%)†‡	72 (52%)*	141 (43%)*	<0.001
CT - Fibrosis (by severity)	†‡	*‡	*†	<0.001
Mild	0 (0%)	54 (39%)	28 (8.6%)	
Moderate	0 (0%)	9 (6.5%)	44 (14%)	
None	197 (100%)	66 (48%)	184 (57%)	
Severe	0 (0%)	0 (0%)	48 (15%)	
Unknown	0 (0%)	9 (6.5%)	21 (6.5%)	
CT - Emphysema	0 (0%)†‡	98 (71%)*	204 (63%)*	<0.001
CT - Emphysema (by severity)	†‡	*‡	*†	<0.001
Mild	0 (0%)	48 (35%)	29 (8.9%)	
Moderate	0 (0%)	38 (28%)	91 (28%)	
None	197 (100%)	40 (29%)	121 (37%)	
Severe	0 (0%)	5 (3.6%)	64 (20%)	
Unknown	0 (0%)	7 (5.1%)	20 (6.2%)	
CT - CPFE	0 (0%)†‡	32 (23%)*	69 (21%)*	<0.001
mPAP (mmHg)	56 (13) †‡	49 (9)* ‡	42 (10)* †	<0.001
mRAP (mmHg)	11.4 (6.1) ‡	11.3 (5.3) ‡	9.0 (5.1)* †	<0.001
PAWP (mmHg)	10.6 (3.1) ‡	11.1 (3.6)	11.8 (4.2)*	0.004
Cardiac output (L/min)	4.46 (1.74) ‡	4.10 (1.41)	5.00 (1.64)	<0.001
Cardiac index (L/min x m^-2)	2.39 (0.87) ‡	2.23 (0.71) ‡	2.73 (0.87)* †	<0.001
PVR (Wood Units)	11.6 (5.7) †‡	10.2 (4.4)* ‡	7.0 (4.1)* †	<0.001
SvO2 %	62 (10) ‡	59 (8) ‡	65 (8)* †	<0.001
FEV_1 % predicted	83 (17)* ‡	83 (20) ‡	60 (25)* †	<0.001
FEV_1 severity	‡	‡	*†	<0.001
Normal (>80% predicted)	114 (61%)	73 (54%)	72 (24%)	

Mild (70-80% predicted)	29 (16%)	24 (18%)	34 (11%)	
Moderate (50-70% predicted)	39 (21%)	31 (23%)	69 (23%)	
Severe (<50% predicted)	5 (2.7%)	6 (4.5%)	129 (42%)	
FEV_1 / FVC (%)	74 (10) †‡	67 (10)* ‡	61 (18)* †	<0.001
DLco % predicted	53 (19) †‡	32 (15)* ‡	28 (14)* †	<0.001

Data are presented as number (percentage) or mean (standard deviation). Between-group comparisons performed using one-Way ANOVA with Bonferroni Post-Hoc Correction. Difference between groups noted: * significant difference to IPAH-noLD, † significant difference to IPAH-LD, ‡ significant difference to PH-CLD. Abbreviations: IPAH-LD, Idiopathic Pulmonary Arterial Hypertension with lung disease, PH-CLD, PH due to chronic lung disease and/or hypoxia, CT – Computed Tomography, WHO – World Health Organisation, CPFE – Combined Pulmonary Fibrosis and Emphysema, mPAP – mean pulmonary arterial pressure, mRAP – mean right atrial pressure, PAWP – pulmonary arterial wedge pressure, PVR – pulmonary vascular resistance, SvO_2 – mixed venous oxygen saturation. FEV_1 – forced expiratory volume in 1 second, FVC – forced vital capacity, DLco – diffusing capacity of carbon monoxide.

Table A3: Survival of patients with IPAH-noLD, IPAH-LD and PH-CLD

Characteristic	1 Year	2 Year	3 Year	4 Year	5 Year
IPAH-noLD	92%	85%	82%	78%	70%
IPAH-LD	87%	60%	41%	29%	21%
PH-CLD	69%	46%	36%	28%	20%

Abbreviations: IPAH-noLD, Idiopathic Pulmonary Arterial Hypertension with no lung disease; IPAH-LD, Idiopathic Pulmonary Arterial Hypertension with lung disease; PH-CLD, PH due to chronic lung disease and/or hypoxia.
